# Water Extract of *Acer tegmentosum* Reduces Bone Destruction by Inhibiting Osteoclast Differentiation and Function

**DOI:** 10.3390/molecules19043940

**Published:** 2014-04-01

**Authors:** Hyunil Ha, Ki-Shuk Shim, Taesoo Kim, Hyosun An, Chung-Jo Lee, Kwang Jin Lee, Jin Yeul Ma

**Affiliations:** KM-Based Herbal Drug Development Group, Korea Institute of Oriental Medicine, Daejeon 305-811, Korea

**Keywords:** *Acer tegmentosum*, osteoclast, bone, RANKL

## Abstract

The stem of *Acer tegmentosum* has been widely used in Korea for the treatment of hepatic disorders. In this study, we investigated the bone protective effect of water extract of the stem of *Acer tegmentosum* (WEAT). We found that WEAT inhibits osteoclast differentiation induced by receptor activator of nuclear factor-κB ligand (RANKL), an essential cytokine for osteoclast differentiation. In osteoclast precursor cells, WEAT inhibited RANKL-induced activation of JNK, NF-κB, and cAMP response element-binding protein, leading to suppression of the induction of c-Fos and nuclear factor of activated T cells cytoplasmic 1, key transcription factors for osteoclast differentiation. In addition, WEAT inhibited bone resorbing activity of mature osteoclasts. Furthermore, the oral administration of WEAT reduced RANKL-induced bone resorption and trabecular bone loss in mice. Taken together, our study demonstrates that WEAT possesses a protective effect on bone destruction by inhibiting osteoclast differentiation and function.

## 1. Introduction

Bone is constantly remodeled through the removal of old bone by osteoclasts and the replacement of new bone by osteoblasts. Osteoclasts are multinucleated bone resorbing cells that are differentiated from monocyte/macrophage-lineage hematopoietic progenitor cells [1]. The TNF ligand superfamily member, receptor activator of nuclear factor NF-κB ligand (RANKL), which is expressed in cells of osteoblastic lineage and lymphoid tissue, is a pivotal factor for the formation, function, and survival of osteoclasts [2,3]. Osteoprotegerin (OPG) functions as a soluble decoy receptor for RANKL and competes with RANK for RANKL binding, thereby preventing RANKL-induced osteoclastogenesis [2].

Upon binding of RANKL to RANK expressed on osteoclast precursors, TNF receptor-associated factors are recruited to the intracellular domain of RANK and activate downstream signaling pathways including mitogen-activated protein (MAP) kinases and NF-κB, and cAMP response element-binding protein (CREB), which leads to the induction of osteoclastogenic transcription factors such as c-Fos and nuclear factor of activated T cells cytoplasmic 1 (NFATc1) [4,5,6,7,8,9,10].

Previous studies have demonstrated that the RANKL-RANK-OPG axis is not only important for normal bone remodeling but also plays a crucial role in pathological bone destruction [11]. As increased RANKL activity is observed in patients with bone destructive diseases such as osteoporosis, rheumatoid arthritis, and bone metastases, the RANKL-RANK-OPG axis is considered to be the most relevant therapeutic target for bone destructive diseases [11].

There is accumulating evidence that common vegetables and herbal products can be one promising approach for the prevention and treatment of osteoporosis [12,13]. To find medicinal herbs with potential bone protective effects, we investigated the effects of various herbal extracts on RANKL-induced osteoclast differentiation from its precursor cells and found that water extract of the stem of *Acer tegmentosum* (WEAT) has relatively strong inhibitory activity. *Acer* *tegmentosum* is a type of deciduous tree distributed in the Northeast Asia including Korea, Russia, and northern areas of China. In Korea, the stem of *Acer tegmentosum* has been traditionally used for the treatment of hepatic disorders, including hepatitis, hepatic cancer, and hepatic cirrhosis. Previous studies have shown that extracts of the stem of *Acer tegmentosum* possess various pharmacological properties including anti-inflammatory, anti-gastrophatic, anti-adipogenic, anti-cancer, and anti-oxidant effects [14,15,16,17,18]. However, the effects of *Acer tegmentosum* on bone metabolism have not been studied. In the present study, we investigated the anti-osteoclastogenic effect and action mechanism of WEAT. Using a murine model of bone destruction by RANKL, its bone protective effect was also examined. 

## 2. Results and Discussion

### 2.1. WEAT Inhibits RANKL-Induced Osteoclast Differentiation

**Figure 1:**
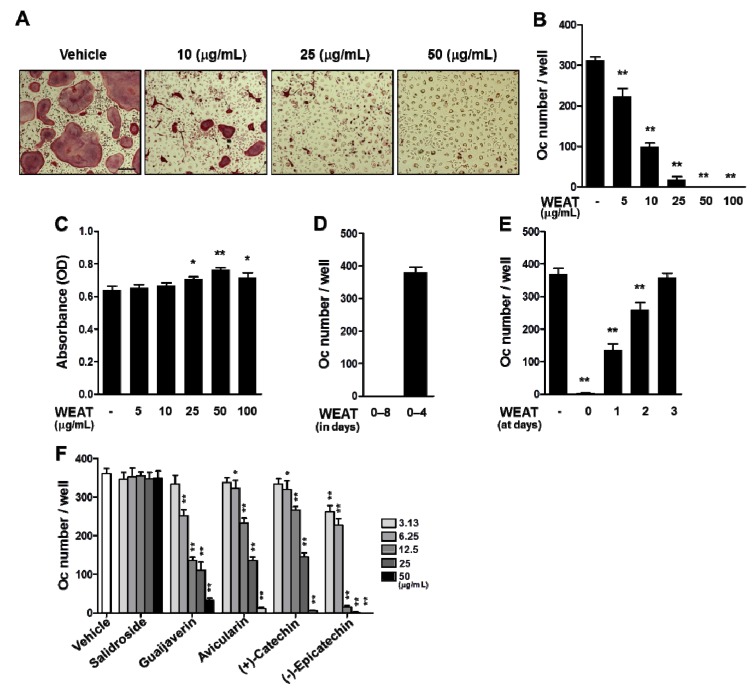
Effect of WEAT on RANKL-induced osteoclast differentiation in BMMs. (**A**,**B**) BMMs were cultured with vehicle (distilled water) or WEAT in the presence of M-CSF (60 ng/mL) and RANKL (100 ng/mL) for 4 days. (**A**) Cells were fixed and stained for TRAP. Scale bar represents 200 μm. (**B**) The number of osteoclasts (Oc) was counted. (**C**) The viability of BMMs was determined by Cell Counting Kit-8 assay in the presence of M-CSF and WEAT. (**D**) BMMs were cultured with WEAT (50 μg/mL) in the presence of M-CSF and RANKL, and WEAT was kept in the culture or withdrawn on day 4 by medium change. The number of osteoclasts was counted on day 8. (**E**) BMMs were cultured in the presence of M-CSF and RANKL for 4 days. WEAT was added to the medium at the indicated days, and the number of osteoclasts was counted on day 4. (**F**) BMMs were cultured with vehicle (dimethyl sulfoxide) or the indicated compounds in the presence of M-CSF and RANKL for 4 days, and the number of osteoclasts was counted. * *p* < 0.05; ** *p* < 0.01 *versus* vehicle-treated control.

In the presence of M-CSF, RANKL is able to induce the differentiation of precursor cells, such as bone marrow-derived macrophages (BMMs), into osteoclasts without osteoclast-supporting cells [2]. To determine whether WEAT affects osteoclast differentiation, we first examined the effect of WEAT on RANKL-induced osteoclast differentiation from its precursor cells, BMMs. Treatment of BMMs with M-CSF and RANKL for 4 days induced tartrate-resistant acid phosphatase (TRAP)-positive multinucleated osteoclasts. When WEAT was added to the cultures at the same time as RANKL, a dose-dependent inhibition of osteoclast differentiation was observed, with nearly complete inhibition of osteoclast differentiation at 50 μg/mL (Figure 1A,B). WEAT did not negatively affect the viability of BMMs at all concentrations tested. It slightly increased cell proliferation of BMMs at 25–100 μg/mL (Figure 1C). These results suggest that the inhibitory effect of WEAT on osteoclast differentiation is not due to cellular toxicity or cell proliferation. The inhibitory action of WEAT was reversible, because withdrawal of WEAT from the cultures on day 4 after its treatment restored osteoclast differentiation (Figure 1D). We next investigated at which stage WEAT inhibits osteoclast differentiation. WEAT was added to BMM cultures simultaneously with RANKL (day 0) or after 1, 2, or 3 days. Inhibition of osteoclast differentiation was maximal when WEAT was added on day 0. The anti-osteoclastogenic activity dramatically decreased when WEAT was added 1 day after RANKL stimulation or thereafter (Figure 1E). These results suggest that WEAT targets the early stage of RANKL-induced osteoclast differentiation. Several flavonoids including quercetin glycosides and (+)-catechin, phenolic glycosides, and steroidal glycosides have been isolated from methanol extract from *Acer tegmentosum* [16,19,20]. We previously found salidroside to be one of the main components of WEAT [21]. It has been shown that salidroside possesses multiple pharmacological properties including hepato-protective, anti-inflammatory, anti-oxidant, lipid lowing, and neuroprotective effects [22,23,24,25,26]. In addition, recent studies have reported that salidroside protects against ovariectomy-induced osteoporosis through inhibition of oxidative stress-induced osteoblast dysfunction and stimulation of osteoblast differentiation [27,28]. We investigated the effect of salidroside on osteoclast differentiation and found that salidroside does not affect RANKL-induced osteoclast differentiation at 3.13–50 μg/mL concentrations (Figure 1F). In an effort to find the active components responsible for the anti-osteoclastogenic effect of WEAT, we isolated four flavonoids (guaijaverin, avicularin, (+)-catechin, and (−)-epicatechin) from WEAT by chromatographic separation. The structures of these compounds were determined using nuclear magnetic resonance and liquid chromatography-mass spectrometry (data not shown). These four flavonoids inhibited RANKL-induced osteoclast differentiation in BMMs in a dose-dependent manner (Figure 1F). These results suggest that the anti-osteoclastogenic effect of WEAT might result from the complementary effects of its active components including these four flavonoids. 

### 2.2. WEAT Inhibits RANKL-Induced c-Fos and NFATc1 Expression in Osteoclast Precursor Cells

**Figure 2:**
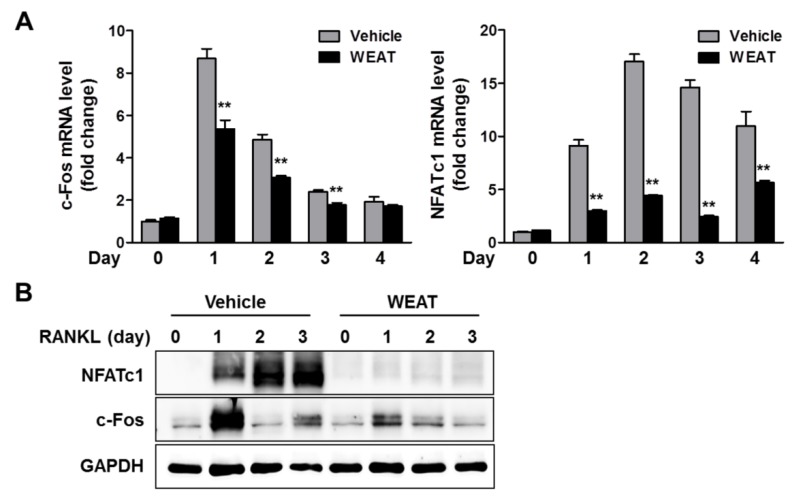
Effect of WEAT on RANKL-induced c-Fos and NFATc1 expression in BMMs. BMMs were cultured with vehicle or WEAT (50 μg/mL) in the presence of M-CSF (60 ng/mL) and RANKL (100 ng/mL) for the indicated days. (**A**) mRNA expression levels of c-Fos and NFATc1 were determined by real-time quantitative polymerase chain reaction (qPCR). ** *p* < 0.01 *versus* vehicle-treated control. (**B**) Protein expression levels of c-Fos and NFATc1 were determined by western blot analysis. Total GAPDH was used as a loading control.

Because WEAT could inhibit RANKL-induced osteoclast differentiation via directly acting on osteoclast precursor cells, we investigated the effect of WEAT on the expression of key transcription factors required for osteoclast differentiation. RANKL induces NFATc1 expression at a transcription level in osteoclast precursors, and NFATc1-deficient cells fail to differentiate osteoclasts in response to RANKL. In addition, ectopic overexpression of NFATc1 in osteoclast precursor cells induces osteoclast differentiation in the absence of RANKL [6]. Therefore, NFATc1 is considered to be a master transcription factor for osteoclast differentiation. RANKL strongly increased NFATc1 mRNA and protein expression in BMMs, which was markedly inhibited by WEAT treatment (Figure 2A,B). The transcription factor c-Fos is also essential for osteoclast differentiation and functions as a key upstream activator of NFATc1 for osteoclast differentiation [29]. We next examined the effect of WEAT on RANKL-induced c-Fos expression and found that WEAT inhibits RANKL-induced c-Fos mRNA and protein expression in BMMs (Figure 2A,B). These results suggest that WEAT blunts RANKL-induced NFATc1 expression by suppressing c-Fos induction, thereby inhibiting osteoclast differentiation. 

### 2.3. WEAT Inhibits RANKL-Induced JNK, NF-κB, and CREB Activation

**Figure 3:**
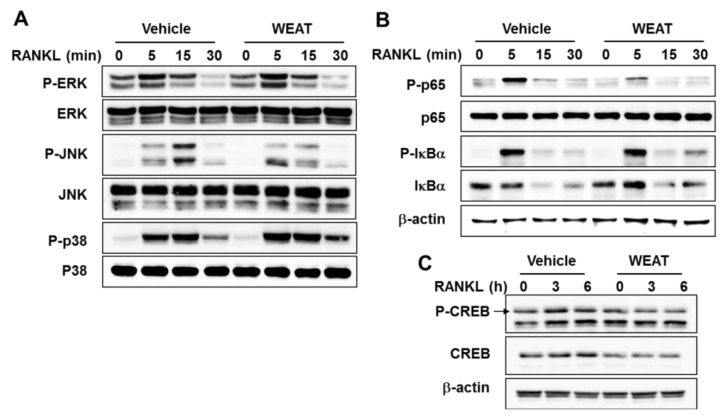
Effect of WEAT on RANKL-Induced MAP kinase, NF-κB, and CREB activation in BMMs. (**A**–**C**) BMMs were pretreated with WEAT (50 μg/mL) for 3 h and then stimulated with RANKL (100 ng/mL) for the indicated times. The total and phosphorylated levels of the indicated proteins were determined by western blot analysis. β-actin was used as a loading control.

Binding of RANKL to RANK activates ERK, JNK, and p38 MAP kinases in osteoclast precursor cells, and these MAP kinases are involved in c-Fos and NFATc1 induction as well as osteoclast differentiation [7,8,9,30]. To gain more insights into the anti-osteoclastogenic effect of WEAT, we examined the effect of WEAT on the activation of MAP kinases. The phosphorylation of ERK, JNK, and p38 MAP kinases by RANKL increased within 5 min, reached the maximum at 5 or 15 min, and then decreased thereafter. WEAT decreased RANKL-induced phosphorylation of JNK but not ERK and p38 (Figure 3A). 

Mice lacking both of the NF-κB p50 and p52 subunits develop osteoporosis due to defective osteoclast development [31]. In addition, it was reported that NF-κB p50/p52 double knockout osteoclast precursors fail to induce c-Fos and NFATc1 expression in response to RANKL, and overexpression of c-Fos rescues the defect in osteoclast differentiation in NF-κB p50/p52 double knockout osteoclast precursors [5]. The classical NF-κB signaling pathway involves IκB kinase (IKK) complex-mediated phosphorylation and degradation of IκBα which allows NF-κB heterodimer containing the p65 and p50 subunits to translocate to the nucleus and activate transcription of target genes [32]. In osteoclast precursors, inhibition of the classical NF-κB signaling pathway by IKKβ deficiency or a cell-permeable peptide inhibitor of IKK complex has been shown to inhibit RANKL-induced NF-κB activation and osteoclastogenesis [33,34,35]. Thus, we examined whether WEAT affects RANKL-induced activation of the classical NF-κB pathway. WEAT did not affect RANKL-induced phosphorylation and subsequent degradation of IκBα in BMMs (Figure 3B). In addition to IκBα, p65 modifications including phosphorylation and acetylation also affect NF-κB transcriptional activity [32]. It has been reported that phosphorylation of p65 at Ser536 in the transactivation domain increases its transcriptional activity [36,37]. In addition, p65 phosphorylation at Ser536 has been shown to influence transcription of distinct sets of NF-κB responsive genes that is independent of IκBα [38]. Consistent with previous reports [8,39], RANKL increased phosphorylation of p65 at Ser536 in BMMs, and WEAT inhibited the phosphorylation of p65 by RANKL (Figure 3B). These results suggest that WEAT might affect RANKL-induced NF-κB activation by inhibiting p65 phosphorylation at Ser536. However, whether the decrease in p65 phosphorylation at Ser536 is involved in the anti-osteoclastogenic effect of WEAT remains to be elucidated. 

The calcium/calmodulin dependent kinase IV-CREB pathway also plays an important role in the regulation of c-Fos and NFATc1 induction during osteoclast differentiation [10]. In addition, it has been shown that 70% ethanol extract of *Acer* *tegmentosum* suppresses LPS-induced CREB activation in RAW264.7 cells [14]. Thus, we examined whether WEAT affects RANKL-induced CREB activation. Treatment of BMMs with WEAT decreased CREB protein expression and blunted RANKL-induced CREB phosphorylation (Figure 3C). Collectively, our findings suggest that WEAT inhibits c-Fos expression during osteoclast differentiation, at least in part, by suppressing RANKL-induced JNK, NF-κB, and CREB activation. 

### 2.4. WEAT Inhibits Bone Resorbing Activity of Osteoclasts

**Figure 4:**
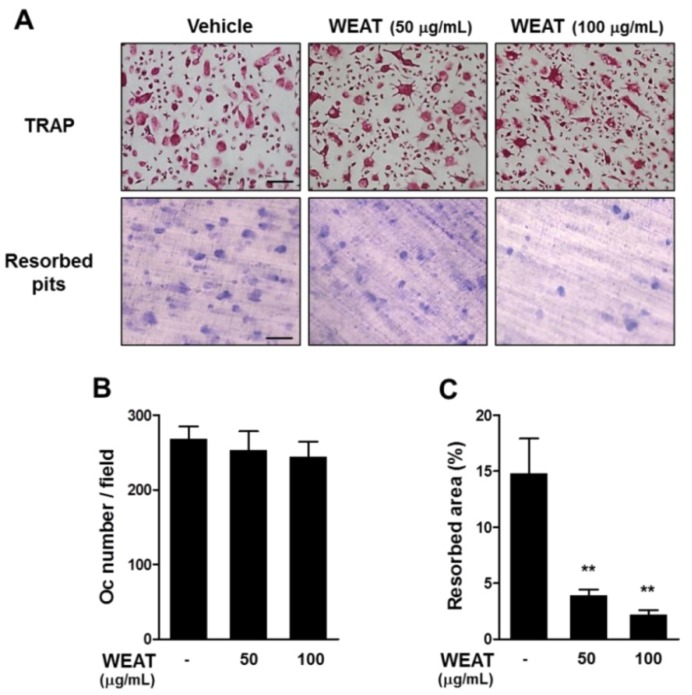
Effect of WEAT on osteoclast function. Mature osteoclasts were plated on dentin slices, preincubated with or without WEAT for 3 h, and then treated with RANKL (100 ng/mL) for 16 h. (**A**) TRAP staining (upper panel) and resorbed pits by osteoclasts (down panel). Scale bars represent 100 μm. (**B**) The number of osteoclasts (Oc). (**C**) The total area of resorbed pits. ** *p* < 0.01 *versus* vehicle-treated control.

Bone resorbing activity is the unique ability of osteoclasts. When attached to bone matrix, mature osteoclasts polarize their membrane to bone surface and secret protons and lytic enzymes that degrade bone matrix in a sealed compartment [1]. We next investigated whether WEAT affects bone resorbing activity of osteoclasts. Mature osteoclasts were obtained from BMM cultures treated with M-CSF and RANKL. When mature osteoclasts were cultured on dentin slices in the presence of M-CSF and RANKL for 16 h, resorption pits were formed on the slices. Pretreatment with 100 μg/mL of WEAT decreased the total resorbed area by osteoclasts but did not affect the number of osteoclasts (Figure 4A–C), suggesting that WEAT inhibits bone resorbing activity of mature osteoclasts. 

### 2.5. WEAT Reduces RANKL-Induced Bone Destruction in Mice

**Figure 5:**
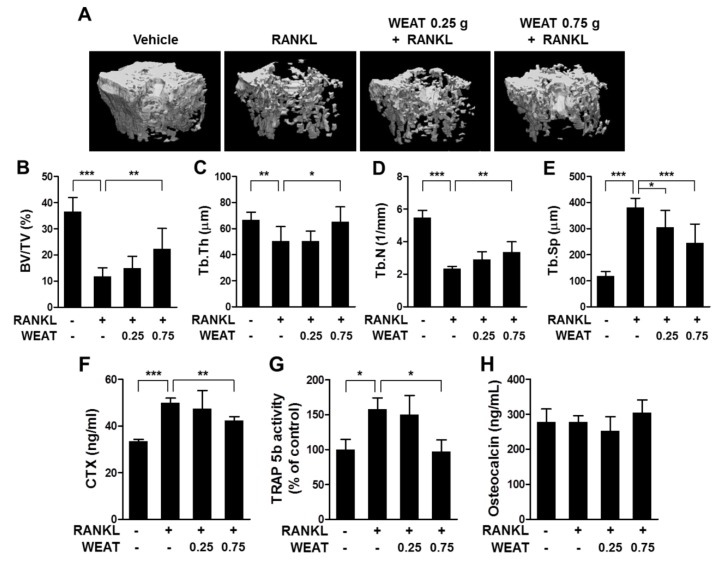
Effect of WEAT on RANKL-induced bone destruction. Mice were orally administrated with WEAT (0.25 and 0.75 g/kg) at 12 h intervals for 5 consecutive days, and RANKL (1 mg/kg) was injected intraperitoneally on days 3 and 4. Femora and blood samples were collected on day 6. Three-dimensional micro-CT images of trabecular bone (**A**), trabecular bone volume/tissue volume (BV/TV, **B**), trabecular thickness (Tb.Th, **C**), trabecular number (Tb.N, **D**), and trabecular separation (Tb.Sp, **E**) at the distal femoral metaphysis. (**F**) Serum CTX levels. (G) Serum TRAP 5b activity. (**H**) Serum osteocalcin levels. * *p* < 0.05; ** *p* < 0.01; *** *p* < 0.001.

Having found that WEAT inhibits osteoclast differentiation as well as function of mature osteoclasts, we next asked whether WEAT has a protective effect on bone destruction associated with enhanced osteoclastic bone resorption. We used a murine model of bone destruction by RANKL injection. It was reported that intraperitoneal administration of RANKL rapidly induces bone destruction by stimulating osteoclast differentiation and function [40]. Consistent with this, intraperitoneal injections of RANKL into mice resulted in a severe trabecular bone loss at the distal femoral metaphysis (Figure 5A). Micro-computed tomography (micro-CT) analysis revealed a marked decrease in trabecular bone volume, thickness, and number, with an increase in trabecular separation in the femoral metaphysis of RANKL-injected mice (Figure 5B–E). Oral administration of WEAT (0.75 g/kg) reduced RANKL-induced bone loss and alterations of trabecular architecture, while 0.25 g/kg of WEAT attenuated only the change in trabecular separation (Figure 5A–E). We next investigated the changes in serum C-terminal cross-linked telopeptide of type I collagen (CTX) levels, TRAP isoenzyme 5b (TRAP 5b) activity, and osteocalcin levels, which can be used for markers of bone resorption, osteoclast numbers, and bone formation, respectively [41]. Serum CTX levels and TRAP 5b activity were elevated following RANKL injections, which was inhibited by administration of 0.75 g/kg of WEAT (Figure 5F,G). No significant changes in serum osteocalcin levels were observed between all groups (Figure 5H). Collectively, these results suggest that the bone protective effect of WEAT is mainly due to inhibition of osteoclastic bone resorption rather than stimulation of osteoblastic bone formation. 

## 3. Experimental

### 3.1. Reagents and Antibodies

α-Modified minimal essential medium (α-MEM), fetal bovine serum, and antibiotics (100 U/mL penicillin and 100 μg/mL streptomycin) were purchased from Thermo Fisher Scientific Inc. (Rockford, IL, USA). Recombinant M-CSF and RANKL were obtained as described previously [42]. Antibodies against phospho-ERK1/2 (Thr202/Tyr204), ERK, phospho-JNK1/2 (Thr183/Tyr185), JNK, phospho-p38 (Thr180/Tyr182), p38, phospho-IκBα (Ser32), IκBα, phospho-p65 (Ser536), and p65, phospho-CREB (Ser133), and CREB were obtained from Cell Signaling Technology (Danvers, MA, USA). Antibodies against NFATc1, c-Fos, and GAPDH were purchased from Santa Cruz Biotechnology (Santa Cruz, CA, USA). Antibody against β-actin was obtained from Sigma-Aldrich (St. Louis, MO, USA).

### 3.2. Preparation of WEAT

Air-dried stem of *Acer tegmentosum* were obtained from Yeongcheon Oriental Herbal Market (Yeongcheon, Korea). A voucher specimen (registration number: #134) was deposited at the herbarium of the KM-Based Herbal Drug Development Group, Korea Institute of Oriental Medicine. The stem of *Acer tegmentosum* (3.0 kg) was extracted with 30 L of filtered tap water at 115 °C for 3 h. The water extract was filtered with standard sieves (106 μm; Restsch, Haan, Germany) and then lyophilized. The lyophilized powder (yield: 3.4% of dry weight) was re-suspended in distilled water and centrifuged at 10,000 ×*g* for 5 min to prepare WEAT. After being filtered through a 0.2 μm filter, WEAT was used for *in vitro* experiments. 

### 3.3. Cell Culture and Osteoclast Differentiation

To obtain bone marrow cells, the bone marrow cavity of tibiae and femora of 6- to 7-week-old male ICR mice (Orient Bio Inc., Seoul, Korea) were flushed with α-MEM, and red blood cells were removed with a red blood cell lysis buffer (0.01 mM EDTA, 10 mM KHCO_3_, and 155 mM NK_4_Cl, pH 7.3). BMMs were prepared from mouse bone marrow cells as described previously [43] and cultured in α-MEM complete medium containing 10% fetal bovine serum and antibiotics in the presence of M-CSF (60 ng/mL). For osteoclast differentiation, BMMs (1 × 10^4^ cells/well) were cultured in α-MEM complete medium supplemented with M-CSF (60 ng/mL) and RANKL (100 ng/mL) for 4 days in a 96-well plate. WEAT was added to the cultures simultaneously with M-CSF and RANKL at day 0 or after 1, 2, or 3 days. Cultures were replenished with fresh medium and treatments every 3 days. After incubation for 4 or 8 days, osteoclast differentiation was analyzed by staining for TRAP activity as described below. TRAP-positive multinucleated cells containing more than three nuclei and larger than 100 μm in diameter were counted as osteoclasts.

### 3.4. TRAP Assay

TRAP staining were performed as described previously [42]. Briefly, cells were fixed in 10% neutral buffered formalin and then permeabilized with 0.1% Triton X-100 in PBS. To identify osteoclasts, cells were stained using TRAP buffer (50 mM sodium tartrate and 0.12 M sodium acetate, pH 5.2) with naphthol AS-MX phosphate (0.1 mg/mL; Sigma-Aldrich) and fast red violet LB salt (0.5 mg/mL; Sigma-Aldrich) and photographed under a light microscope (Magnification ×100). Serum TRAP 5b activity was determined using the fluorogenic substrate, naphthol ASBI phosphate (Sigma-Aldrich), as previously reported with slight modifications [44]. Serum was incubated with TRAP 5b reaction buffer (2.5 mM naphthol AS-BI phosphate, 50 mM sodium tartrate and 0.12 M sodium acetate, 2% NP-40, and 1% ethylene glycol monomethyl ether, pH 6.1) for 30 min at 37 °C. The enzymatic reaction was stopped by adding 0.1 M NaOH, and fluorescence was measured at an excitation wavelength of 405 nm and an emission wavelength of 520 nm.

### 3.5. Cell Viability Assay

BMMs (1 × 10^4^ cells/well) were cultured with M-CSF (60 ng/mL) for 2 days in a 96-well plate. The viability of BMMs was evaluated with Cell Counting Kit-8 assay (Dojindo Molecular Technologies Inc., Rockville, MD, USA). The absorbance was measured at 450 nm with 650 nm as reference using a microplate reader.

### 3.6. Bone Resorption Assay

BMMs (2 × 10^6^ cells) were cultured in α-MEM complete medium supplemented with M-CSF (60 ng/mL) and RANKL (100 ng/mL) for 3 days in a 10-cm culture dish. The cells were collected by using cell dissociation buffer (Invitrogen, Carlsbad, CA, USA) and further cultured with M-CSF and RANKL on a collagen gel (Cellmatrix type I-A, Nitta Gelatin Inc., Osaka, Japan) for 3 days to generate osteoclasts. Collagen was digested with 0.2% collagenase (Sigma-Aldrich), and osteoclasts were placed on dentin slices in 96-well plates in the presence of M-CSF (60 ng/mL), allowed to settle for 2 h, preincubated with or without WEAT for 3 h, and then treated with RANKL (100 ng/mL) for an additional 16 h. Cells on dentin slices were stained for TRAP activity. To determine bone resorbing activity, dentin slices were stained with 2% toluidine blue after removing cells with 1% ammonium hydroxide. Osteoclasts and resorbed pits were photographed at a magnification of 100×, and the number of osteoclasts and the resorption area were analyzed in 3 randomly selected fields of each dentine slice using Image J software.

### 3.7. qPCR Analysis

Total RNA was isolated with RNA-spin total RNA Extraction Kit (Bioneer, Daejeon, Korea), and cDNA was synthesized from 1 μg of total RNA with AccuPower RT-PreMix (Bioneer). SYBR green-based qPCR amplification was performed on the Applied Biosystems 7500 Real-Time PCR System (Foster City, CA, USA) with cDNA diluted to 1:3, 10 pmol of primers, and AccuPower GreenStar qPCR Master Mix (Bioneer). The primer sequences used were previously described [43]. All reactions were run in triplicate, and data were analyzed using the 2^−∆∆CT^ method. Hypoxanthine phosphoribosyltransferase was used as an internal control.

### 3.8. Western Blot Analysis

BMMs were washed with ice-cold PBS and lysed in RIPA buffer (Millipore, Temecula, CA, USA) with protease-inhibitor and phosphatase-inhibitor cocktail tablets (Roche Applied Science, Indianapolis, IL, USA). The whole-cell extracts were vigorously vortexed and then centrifuged at 10,000 ×*g* for 10 min. Protein concentrations in the supernatants were determined using a DC Protein Assay Kit (Bio-Rad, Hercules, CA, USA). Equal amounts of proteins (30 μg) were subjected to sodium dodecyl sulfate-polyacrylamide gel electrophoresis, transferred to a polyvinylidene fluoride membrane, and then immunoblotted with the indicated antibodies. Chemiluminescent signals were detected using a Luminescent Image Analyzer LAS-4000 (Fuji Photo Film Co., Tokyo, Japan) with SuperSignal West Femto Maximum Sensitivity Substrate (Thermo Fisher Scientific Inc.). 

### 3.9. Animals and RANKL-Induced Osteoporosis Model

Animal experiments were performed according to the Guide for the Care and Use of Laboratory Animals of the National Institutes of Health. The experimental protocols were approved by the Institutional Animal Care and Use Committee at Korea Institute of Oriental Medicine. After acclimatization for 1 week, 7-week-old male ICR mice (7 mice/group) were orally administered with distilled water or WEAT (0.25 and 0.75 g/kg of body weight) twice daily for 5 days. RANKL (1 mg/kg of body weight) or PBS was intraperitoneally injected on days 3 and 4. On day 6, fasting blood samples and the right femora were collected. Serum levels of CTX and osteocalcin were measured using a RatLaps EIA kit (Immunodiagnostic Systems Inc., Fountain Hills, AZ, USA) and a mouse osteocalcin EIA kit (Biomedical Technologies Inc., Stoughton, MA, USA), respectively. Micro-CT scanning of the distal femora was performed with the SMX-90CT system (Shimadzu, Kyoto, Japan). Scans were integrated into 3D voxel images and reconstructed by the VG Studio MAX 1.2.1 program (Volume Graphics, Heidelberg, Germany). Trabecular bone architecture was assessed at the distal femoral metaphysis within a 2-mm region initiating at the distal edge of the metaphyseal growth plate with TRI/3D-BON (RATOC System Engineering, Kyoto, Japan). 

### 3.10. Statistical Analysis

Data are presented as mean ± SD in *in vitro* experiments and mean ± SEM in *in vivo* experiments. Two-group comparisons were performed with Student’s *t* tests, while multiple-group comparisons were performed with analysis of variance followed by Dunnett’s test. A *p*-value less than 0.05 was considered statistically significant. 

## 4. Conclusions

WEAT inhibited RANKL-induced osteoclast differentiation by suppressing c-Fos and NFATc1 expression through interfering with RANK-mediated signals in osteoclast precursor cells. In addition, WEAT inhibited bone resorbing activity of mature osteoclasts. Consistent with the *in vitro* results, oral administration of WEAT reduced RANKL-induced osteoclastic bone resorption and bone loss *in vivo*. Taken together, our study shows that WEAT reduces bone destruction by inhibiting osteoclast differentiation and function. Since excess osteoclast activity is critical in pathological bone destruction, our results suggest that WEAT may be useful for the prevention and treatment of various bone diseases associated with excessive bone resorption. 

## References

[1] Boyle W.J., Simonet W.S., Lacey D.L. (2003). Osteoclast differentiation and activation. Nature.

[2] Lacey D.L., Timms E., Tan H.L., Kelley M.J., Dunstan C.R., Burgess T., Elliott R., Colombero A., Elliott G., Scully S. (1998). Osteoprotegerin ligand is a cytokine that regulates osteoclast differentiation and activation. Cell.

[3] Kong Y.Y., Yoshida H., Sarosi I., Tan H.L., Timms E., Capparelli C., Morony S., Oliveira-dos-Santos A.J., Van G., Itie A. (1999). OPGL is a key regulator of osteoclastogenesis, lymphocyte development and lymph-node organogenesis. Nature.

[4] Gohda J., Akiyama T., Koga T., Takayanagi H., Tanaka S., Inoue J. (2005). RANK-mediated amplification of TRAF6 signaling leads to NFATc1 induction during osteoclastogenesis. EMBO J..

[5] Yamashita T., Yao Z., Li F., Zhang Q., Badell I.R., Schwarz E.M., Takeshita S., Wagner E.F., Noda M., Matsuo K. (2007). NF-kappaB p50 and p52 regulate receptor activator of NF-kappaB ligand (RANKL) and tumor necrosis factor-induced osteoclast precursor differentiation by activating c-Fos and NFATc1. J. Biol. Chem..

[6] Takayanagi H., Kim S., Koga T., Nishina H., Isshiki M., Yoshida H., Saiura A., Isobe M., Yokochi T., Inoue J. (2002). Induction and activation of the transcription factor NFATc1 (NFAT2) integrate RANKL signaling in terminal differentiation of osteoclasts. Dev. Cell.

[7] Huang H., Chang E.J., Ryu J., Lee Z.H., Lee Y., Kim H.H. (2006). Induction of c-Fos and NFATc1 during RANKL-stimulated osteoclast differentiation is mediated by the p38 signaling pathway. Biochem. Biophys. Res. Commun..

[8] Lee J.H., Jin H., Shim H.E., Kim H.N., Ha H., Lee Z.H. (2010). Epigallocatechin-3-gallate inhibits osteoclastogenesis by down-regulating c-Fos expression and suppressing the nuclear factor-kappaB signal. Mol. Pharmacol..

[9] Ikeda F., Nishimura R., Matsubara T., Tanaka S., Inoue J., Reddy S.V., Hata K., Yamashita K., Hiraga T., Watanabe T. (2004). Critical roles of c-Jun signaling in regulation of NFAT family and RANKL-regulated osteoclast differentiation. J. Clin. Invest..

[10] Sato K., Suematsu A., Nakashima T., Takemoto-Kimura S., Aoki K., Morishita Y., Asahara H., Ohya K., Yamaguchi A., Takai T. (2006). Regulation of osteoclast differentiation and function by the CaMK-CREB pathway. Nat. Med..

[11] Tanaka S., Nakamura K., Takahasi N., Suda T. (2005). Role of RANKL in physiological and pathological bone resorption and therapeutics targeting the RANKL-RANK signaling system. Immunol. Rev..

[12] Banu J., Varela E., Fernandes G. (2012). Alternative therapies for the prevention and treatment of osteoporosis. Nutr. Rev..

[13] Putnam S.E., Scutt A.M., Bicknell K., Priestley C.M., Williamson E.M. (2007). Natural products as alternative treatments for metabolic bone disorders and for maintenance of bone health. Phytother. Res..

[14] Yu T., Lee J., Lee Y.G., Byeon S.E., Kim M.H., Sohn E.H., Lee Y.J., Lee S.G., Cho J.Y. (2010). *In vitro* and *in vivo* anti-inflammatory effects of ethanol extract from Acer tegmentosum. J. Ethnopharmacol..

[15] Liu Q., Shin E., Ahn M.J., Hwang B.Y., Lee M.K. (2011). Anti-adipogenic activity of Acer tegmentosum and its constituent, catechin in 3T3-L1 cells. Nat. Prod. Sci..

[16] Kim S., Hur S.H., Kim K.H., Kim S.G., Whang W.K. (2012). Antioxidant and anti-inflammatory compounds isolated from Acer tegmentosum. J. Med. Plants Res..

[17] Lee S., Woo H. (2010). A study of the inhibitory effect of Acer tegmentosum Max. on fibrogenesis in hepatic stellate cell line T6. Korean J. Orient. Int. Med..

[18] Yoo Y.M., Nam J.H., Kim M.Y., Choi J., Lee K.T., Park H.J. (2009). Analgesic and anti-gastropathic effects of salidroside Isolated from Acer tegmentosum heartwood. Open Bioact. Compound. J..

[19] Tung N.H., Ding Y., Kim S.K., Bae K., Kim Y.H. (2008). Total peroxyl radical-scavenging capacity of the chemical components from the stems of Acer tegmentosum maxim. J. Agric. Food Chem..

[20] Park K.M., Yang M.C., Lee K.H., Kim K.R., Choi S.U., Lee K.R. (2006). Cytotoxic phenolic constituents of Acer tegmentosum maxim. Arch. Pharm. Res..

[21] Hwang Y.H., Park H., Ma J.Y. (2013). *In vitro* and *in vivo* safety evaluation of Acer tegmentosum. J. Ethnopharmacol..

[22] Wu Y.L., Lian L.H., Jiang Y.Z., Nan J.X. (2009). Hepatoprotective effects of salidroside on fulminant hepatic failure induced by D-galactosamine and lipopolysaccharide in mice. J. Pharm. Pharmacol..

[23] Li D., Fu Y., Zhang W., Su G., Liu B., Guo M., Li F., Liang D., Liu Z., Zhang X. (2013). Salidroside attenuates inflammatory responses by suppressing nuclear factor-kappaB and mitogen activated protein kinases activation in lipopolysaccharide-induced mastitis in mice. Inflamm. Res..

[24] Zhang L., Yu H., Sun Y., Lin X., Chen B., Tan C., Cao G., Wang Z. (2007). Protective effects of salidroside on hydrogen peroxide-induced apoptosis in SH-SY5Y human neuroblastoma cells. Eur. J. Pharmacol..

[25] Zhang B.C., Li W.M., Guo R., Xu Y.W. (2012). Salidroside decreases atherosclerotic plaque formation in low-density lipoprotein receptor-deficient mice. Evid. Based Complement. Alternat. Med..

[26] Sheng Q.S., Wang Z.J., Zhang J., Zhang Y.G. (2013). Salidroside promotes peripheral nerve regeneration following crush injury to the sciatic nerve in rats. Neuroreport.

[27] Chen J.J., Zhang N.F., Mao G.X., He X.B., Zhan Y.C., Deng H.B., Song D.Q., Li D.D., Li Z.R., Si S.Y. (2013). Salidroside stimulates osteoblast differentiation through BMP signaling pathway. Food Chem. Toxicol..

[28] Zhang J.K., Yang L., Meng G.L., Yuan Z., Fan J., Li D., Chen J.Z., Shi T.Y., Hu H.M., Wei B.Y. (2013). Protection by salidroside against bone loss via inhibition of oxidative stress and bone-resorbing mediators. PLoS One.

[29] Matsuo K., Galson D.L., Zhao C., Peng L., Laplace C., Wang K.Z., Bachler M.A., Amano H., Aburatani H., Ishikawa H. (2004). Nuclear factor of activated T-cells (NFAT) rescues osteoclastogenesis in precursors lacking c-Fos. J. Biol. Chem..

[30] Kim H.J., Lee Y., Chang E.J., Kim H.M., Hong S.P., Lee Z.H., Ryu J., Kim H.H. (2007). Suppression of osteoclastogenesis by N,N-dimethyl-D-erythro-sphingosine: A sphingosine kinase inhibition-independent action. Mol. Pharmacol..

[31] Iotsova V., Caamano J., Loy J., Yang Y., Lewin A., Bravo R. (1997). Osteopetrosis in mice lacking NF-kappaB1 and NF-kappaB2. Nat. Med..

[32] Hayden M.S., Ghosh S. (2012). NF-kappaB, the first quarter-century: Remarkable progress and outstanding questions. Genes Dev..

[33] Jimi E., Aoki K., Saito H., D'Acquisto F., May M.J., Nakamura I., Sudo T., Kojima T., Okamoto F., Fukushima H. (2004). Selective inhibition of NF-kappa B blocks osteoclastogenesis and prevents inflammatory bone destruction *in vivo*. Nat. Med..

[34] Dai S., Hirayama T., Abbas S., Abu-Amer Y. (2004). The IkappaB kinase (IKK) inhibitor, NEMO-binding domain peptide, blocks osteoclastogenesis and bone erosion in inflammatory arthritis. J. Biol. Chem..

[35] Ruocco M.G., Maeda S., Park J.M., Lawrence T., Hsu L.C., Cao Y., Schett G., Wagner E.F., Karin M. (2005). I{kappa}B kinase (IKK){beta}, but not IKK{alpha}, is a critical mediator of osteoclast survival and is required for inflammation-induced bone loss. J. Exp. Med..

[36] Yang F., Tang E., Guan K., Wang C.Y. (2003). IKK beta plays an essential role in the phosphorylation of RelA/p65 on serine 536 induced by lipopolysaccharide. J. Immunol..

[37] Doyle S.L., Jefferies C.A., O'Neill L.A. (2005). Bruton's tyrosine kinase is involved in p65-mediated transactivation and phosphorylation of p65 on serine 536 during NFkappaB activation by lipopolysaccharide. J. Biol. Chem..

[38] Sasaki C.Y., Barberi T.J., Ghosh P., Longo D.L. (2005). Phosphorylation of RelA/p65 on serine 536 defines an I{kappa}B{alpha}-independent NF-{kappa}B pathway. J. Biol. Chem..

[39] Huang H., Ryu J., Ha J., Chang E.J., Kim H.J., Kim H.M., Kitamura T., Lee Z.H., Kim H.H. (2006). Osteoclast differentiation requires TAK1 and MKK6 for NFATc1 induction and NF-kappaB transactivation by RANKL. Cell Death Differ..

[40] Tomimori Y., Mori K., Koide M., Nakamichi Y., Ninomiya T., Udagawa N., Yasuda H. (2009). Evaluation of pharmaceuticals with a novel 50-hour animal model of bone loss. J. Bone Miner. Res..

[41] Naylor K., Eastell R. (2012). Bone turnover markers: Use in osteoporosis. Nat. Rev. Rheumatol..

[42] Ha H., An H., Shim K.S., Kim T., Lee K.J., Hwang Y.H., Ma J.Y. (2013). Ethanol extract of Atractylodes macrocephala protects bone loss by inhibiting osteoclast differentiation. Molecules.

[43] Lee J.H., Kim H.N., Yang D., Jung K., Kim H.M., Kim H.H., Ha H., Lee Z.H. (2009). Trolox prevents osteoclastogenesis by suppressing RANKL expression and signaling. J. Biol. Chem..

[44] Janckila A.J., Takahashi K., Sun S.Z., Yam L.T. (2001). Naphthol-ASBI phosphate as a preferred substrate for tartrate-resistant acid phosphatase isoform 5b. J. Bone Miner. Res..

